# Pediatric trainees’ engagement in the online nutrition curriculum: preliminary results

**DOI:** 10.1186/1472-6920-14-190

**Published:** 2014-09-16

**Authors:** Kadriye O Lewis, Graeme R Frank, Rollin Nagel, Teri L Turner, Cynthia L Ferrell, Shilpa G Sangvai, Rajesh Donthi, John D Mahan

**Affiliations:** Children’s Mercy Hospital, Department of Pediatrics, University of Missouri Kansas City School of Medicine, Kansas City, MO USA; Cohen Children’s Medical Center, Division of Pediatric Endocrinology, Hofstra North Shore-LIJ School of Medicine, New Hyde Park, New York, USA; The Ohio State University College of Medicine, Office of Evaluation, Curriculum Research and Development, Columbus, Ohio USA; Baylor College of Medicine, Texas Children’s Hospital, Houston, Texas USA; Oregon Health and Science University, Doernbecher Children’s Hospital, Portland, Oregon USA; Nationwide Children’s Hospital, The Ohio State University College of Medicine, Columbus, Ohio USA; Children’s Hospital Los Angeles, Keck School of Medicine of the University of Southern California, Los Angeles, California USA

**Keywords:** Nutrition modules, Interactive modules, Pediatric trainees, Online nutrition curriculum

## Abstract

**Background:**

The Pediatric Nutrition Series (PNS) consists of ten online, interactive modules and supplementary educational materials that have utilized web-based multimedia technologies to offer nutrition education for pediatric trainees and practicing physicians. The purpose of the study was to evaluate pediatric trainees’ engagement, knowledge acquisition, and satisfaction with nutrition modules delivered online in interactive and non-interactive formats.

**Methods:**

From December 2010 through August 2011, pediatric trainees from seventy-three (73) different U.S. programs completed online nutrition modules designed to develop residents’ knowledge of counseling around and management of nutritional issues in children. Data were analyzed using SPSS version 19. Both descriptive and inferential statistics were used in comparing interactive versus non-interactive modules. Pretest/posttest and module evaluations measured knowledge acquisition and satisfaction.

**Results:**

Three hundred and twenty-two (322) pediatric trainees completed one or more of six modules for a total of four hundred and forty-two (442) accessions. All trainees who completed at least one module were included in the study. Two-way analyses of variance (ANOVA) with repeated measures (pre/posttest by interactive/non-interactive format) indicated significant knowledge gains from pretest to posttest (p < 0.002 for all six modules). Comparisons between interactive and non-interactive formats for Module 1 (N = 85 interactive, N = 95 non-interactive) and Module 5 (N = 5 interactive, N = 16 non-interactive) indicated a parallel improvement from the pretest to posttest, with the interactive format significantly higher than the non-interactive modules (p < .05). Both qualitative and quantitative data from module evaluations demonstrated that satisfaction with modules was high. However, there were lower ratings for whether learning objectives were met with Module 6 (p < 0.03) and lecturer rating (p < 0.004) compared to Module 1. Qualitative data also showed that completion of the interactive modules resulted in higher resident satisfaction.

**Conclusions:**

This initial assessment of the PNS modules shows that technology-mediated delivery of a nutrition curriculum in residency programs has great potential for providing rich learning environments for trainees while maintaining a high level of participant satisfaction.

**Electronic supplementary material:**

The online version of this article (doi:10.1186/1472-6920-14-190) contains supplementary material, which is available to authorized users.

## Background

Despite the fact that nutrition plays a critical role in health promotion, disease prevention, and treatment of patients, many practicing physicians do not feel comfortable or adequately prepared to provide nutrition counseling related to the nutrition aspects of diseases or concerns of their patients [1, 2]. Neglect in nutrition instruction and deficiencies go back to the 1950s, if not further [3]. Nutrition education has been underrepresented and inadequate at medical schools and residency programs for many years [1, 4–6]. In 1985, a report from the National Academy of Sciences suggested medical schools provide at least 25 hours of nutrition instruction within their curriculum [7]. However, a recent update of a national survey of 109 medical schools found that the average required contact hours with nutrition education was 19.6 and medical students also indicated that time spent studying nutrition in their curriculum was inadequate [8]. As discussed by Darer et al. [9], 63% of physicians reported inadequate training in the area of nutrition counseling for patients with chronic illnesses. These figures suggest that learners continue to recognize gaps in their nutrition education and it seems that residency programs did not meet their needs. The ultimate result of this deficiency is graduating pediatricians without the nutrition competencies required in medical practice, leaving many uncomfortable discussing the topic with their patients.

In response to such deficiencies, some U.S. medical schools have explored online supplemental education for general nutrition education by using CD-ROMs and other multimedia enhanced courses [1, 5, 10, 11]. The University of North Carolina at Chapel Hill was one of the early adopters and leaders of a CD-ROM-based nutrition curriculum and have been distributing the curriculum to medical schools as part of the Nutrition in Medicine (NIM) program since 1995 [12]. Although the NIM program has been used in online nutrition education, there is no information on whether the physicians completing the module are actually able to apply this knowledge in practice. As an extension to the NIM program, the Nutrition Education for Practicing Physicians (NEPP) program offers online education on specific nutrition topics to promote behavior change in overweight patients (http://www.nutritioninmedicine.net/portal/). Other programs such as Nutrition in Preventive Medicine, an interactive web-based module developed by Edwards and Lasswell [11], incorporate interactive exercises, and the online teaching platform (Blackboard) employs quizzes and posttests as a means of assessing mastery of information. This form of computer-based educational modules may meet the challenges that befall many medical institutions, including lack of time and resources for quality nutrition education [1, 12]. Unfortunately, in reviewing the vast number of programs on nutrition, there is not much evidence regarding pediatric residents’ nutrition training and competencies gained in pediatric nutrition.

In 2008 the Pediatric Nutrition Series (PNS) Program was initiated with the sponsorship of Abbott Nutrition. This sponsorship allowed the PNS working group, medical education professionals interested in pediatric nutrition education, to develop an evidence-based, scientific, unbranded, and clinically relevant online education program. The working group was composed of faculty from six pediatric academic medical centers who developed the educational content and the web site hosting the materials. The PNS project utilized contemporary multimedia and communications technologies to initially offer 10 specific nutrition education modules to pediatric residents and practicing physicians. The goal of the PNS initiative was to strengthen medical nutrition practice by providing a free, comprehensive, online nutrition curriculum with clinically relevant, competence/outcome based education adopted from Moore's 2009 Expanded Outcomes Framework [13] for residents and physicians-in-training. Thus, the purpose of this study was to evaluate pediatric trainees’ engagement, knowledge acquisition, and satisfaction with a series of newly created nutrition modules delivered online in both interactive and non-interactive formats.

## Methods

This study was reviewed by the Institution Review Board Expedited Committee at Nationwide Children’s Hospital, Columbus, Ohio on January 19^th^, 2010. The Committee provided exempt status under 45 CFR 46.101(b)(2).

### Study design and population

This cross-sectional study was an analysis of data from all residents who accessed and completed at least one of six interactive or non-interactive PNS modules between December 2010 and August 2011. We chose to focus on data from the first six modules because the last four modules were of recent vintage with low completion numbers at the time of this report. Using email and direct mailing, pediatric program directors/associate directors from all 193 Pediatric Residency Training Programs in the US were invited to encourage their residents to sign up for the free PNS online modules. A total of 73 pediatric training programs located in 36 different states had residents complete at least one PNS module during the study period.

### Curriculum and development

The PNS working group consists of pediatric program directors, medical educators, online medical education experts, and other pediatric faculty. An overview of each working group member and authors of the PNS education modules are available on the Resident Learning Center website (http://www.residentlearningcenter.com).

The PNS content was designed for pediatric residents based on topics and learning objectives developed by a group of pediatric nutrition experts from the American Board of Pediatrics (ABP) nutrition content specifications. These pediatric nutrition experts did not manage module design, construction or validation. The learning objectives served as a reference for the PNS working group as they identified faculty to present on each topic. Each presenting faculty member controlled her/his own content development without commercial bias.

The PNS consists of ten (10) online, interactive modules (Additional file 1) and supplementary educational materials (case discussion and applied learning activities). The PNS working group leadership was responsible for developing case studies and applied learning activities for each module (Appendix 1). Each module was first constructed and posted in a format with limited interactivity (non-interactive format) and then was subsequently upgraded by members of the PNS working group to a more interactive format. Residents chose to complete either the interactive or non-interactive format based on their own preferences and no resident completed both formats of the same module. These features provided opportunities to contrast the impact of interactive versus non-interactive formats utilizing the same content on residents’ engagement, knowledge acquisition and satisfaction.

### Evaluation methods and instruments

The evaluation process assessed the experience and effectiveness of the interactive versus non-interactive module formats in the following three areas:

1. *Engagement* was determined by the number of modules completed for each content area.
2. *Knowledge acquisition* was determined by short-term knowledge gain measured by pretests versus posttests for content knowledge. These tests were developed by presenting faculty who provided 15–20 questions derived from the presented materials in the form of multiple choice questions and answers for pretest and posttest knowledge assessment. The multiple choice questions were reviewed by a member of the PNS working group for content validity and format before inclusion. Each participant received 10 randomized questions from a question bank for the pretest and another 10 randomized questions from the same question bank for the posttest. The learning management system graded the scores of these tests based upon the percentage of correct answers.
3. *Learner satisfaction* was determined by; a quantitative assessment of each learner for each completed module on accomplishment of learning objectives, organization of the module, and satisfaction with the learning experience, and by qualitative analysis of learners’ comments illustrating a more detailed understanding of the their experiences.

To measure learner satisfaction, a module evaluation questionnaire was designed to evaluate the trainees’ perceived educational needs, perceived accomplishment of learning objectives of each module, and satisfaction level related to the design and content of the modules. It consisted of questions on a five-point Likert scale (Strongly Disagree to Strongly Agree) and open-ended questions about the learner's experiences. The module evaluation tool was designed by one of the authors (KOL), modified after discussion with the PNS working group members, and trialed with a small group (four pediatric chief residents). In addition, four non-PNS working group pediatric faculty educators reviewed this evaluation tool and confirmed face and content validity of it.

### Data collection and analysis

De-identified linked data were abstracted from all completed modules from December 2010 through August 2011. Trainees also completed the module evaluation questionnaire at the completion of each nutrition module.

The module evaluation regarding satisfaction was collected anonymously. Not all participants who took the pretest and posttest responded to the request for feedback on the modules; therefore, the sample size for the number of test-takers and evaluation completion of some modules were different. Because the interactive formats for some modules were introduced later in the nine month period, there were also very different numbers of completed modules and formats across the six modules. Only initial pre/posttest attempts were used in module test performance comparisons in cases where a trainee completed the module more than once.

We analyzed the quantitative data using SPSS version 19. Both descriptive and inferential statistics were used in comparing interactive vs. non-interactive modules. Two-way analyses of variance (ANOVA) with repeated measures (pre/posttest by interactive/non-interactive format) were employed to determine the change between pretest and posttest scores using interactive versus non-interactive modules as a between subjects’ factor. Effect sizes (Cohen’s f^2^) [14] were calculated for the pre/posttest, interactive/non-interactive, and interaction factors for each module. Median learner satisfaction scores across modules were calculated based on grouping data into Likert scale class intervals [15]. Chi-square analyses were used to compare satisfaction ratings between modules.

In addition, qualitative data from the open-ended questions in the module evaluations were analyzed thematically [16] by two coders independently. Each coder read and re-read responses (free text), with the goal of organizing the data into systematic categories by seeking recurring patterns (predominant themes). We compared the data-driven theme categories and sub-themes to obtain the inter-rater agreement between the codings.

## Results

Our study found the following results regarding pediatric trainees’ engagement, change in knowledge and satisfaction with the PNS modules as a learning medium.

### 1. Engagement

Two hundred and thirty-four individuals (221 pediatric residents and 13 neonatal fellows) from 73 pediatric residency programs in 36 states in the U.S. completed one or more of the six modules. There were 323 accessions for non-interactive modules while 119 accessions were for the interactive versions of the modules producing a total of 442 accessions (97% completed three or fewer, 78% completed only one, and four trainees completed all six modules - Table 1).

**Table 1:** **Module percent correct knowledge comparisons: pre-test versus post-test and interactive versus non-interactive including effect sizes**

Modules	Interactive (I)			Non-Interactive (NI)			Repeated measures ANOVA		
	Pre	Post		Pre	Post		Pre/Post	I/NI	Pre/Postby I/NI
	Mean (SD)	Mean (SD)	N	Mean (SD)	Mean (SD)	N	F (p) [f ^2^]	F (p) [f ^2^]	F (p) [f ^2^]
M1	57.53 (16.40)	89.06 (7.50)	85	54.11 (18.82)	84.11 (15.33)	95	**400.41 (.001)**	**6.23 (.02)**	0.25 (.62)
							[2.25]	[.035]	[.001]
M2	72.00 (13.17)	85.00 (14.34)	10	66.45 (15.31)	82.41 (12.87)	141	**29.61 (.001)**	1.16 (.28)	0.31 (.58)
							[.199]	[.008]	[.002]
M3	78.00 (10.33)	91.00 (15.95)	10	71.25 (16.76)	79.17 (21.04)	24	**10.97 (.002)**	2.53 (.12)	0.65 (.43)
							[.342]	[.079]	[.002]
M4	52.00 (16.43)	86.00 (5.48)	5	53.33 (20.40)	71.67 (22.14)	30	**30.90 (.001)**	0.56 (.46)	2.77 (.11)
							[.938]	[.017]	[.083]
M5	46.00 (8.94)	80.00 (0.00)	5	40.00 (14.61)	60.00 (17.13)	16	**39.67 (.001)**	**4.65 (.05)**	2.67 (.12)
							[2.09]	[.245]	[.140]
M6	67.50 (9.57)	82.50 (5.00)	4	52.35 (13.48)	74.12 (17.34)	17	**14.99 (.001)**	3.22 (.09)	0.51 (.49)
							[.789]	[.170]	[.027]

M = Module.

f^2^ = Cohen’s f^2^ effect size (small effect = .02, medium effect = .15, large effect = .35).

Significant differences (p < 0.05) in **Bold**.

### 2. Knowledge Acquisition

In an analysis of the data from both interactive and non-interactive modules, repeated measures ANOVAs indicated significant knowledge gain from pretest to posttest for both interactive and non-interactive formats for each module.

Fewer trainees completed interactive Modules 2 through 6 than completed interactive Module 1. Comparisons between interactive and non-interactive Module 1 formats (N = 85 interactive, N = 95 non-interactive) indicated parallel improvement from the pretest to the posttest with the interactive module significantly higher (p < .02) than the non-interactive module. There was a similar significant (p < .05) parallel difference in knowledge improvement between the interactive and non-interactive versions for Module 5.

The pre/post effect size was consistently large for each module (.789 for Module 6 to 2.25 for Module 1) except for a medium-to-large effect size (.199) for Module 2 (see Table 1). Most of the interactive/non-interactive and interaction factors had small effect sizes. Only the interactive/non-interactive Module 5 (.245) and Module 6 (.170) as well as the Module 5 interaction (.140) had a medium or larger effect size.

### 3. Learner satisfaction

Learner satisfaction was determined by an anonymous, quantitative assessment of each learner for each completed module on accomplishment of learning objectives, organization of the module and satisfaction with the learning experience, as well as by a qualitative analysis of learners’ comments to provide more detailed understanding of the learners’ experiences.

A. Results from the quantitative analysis

Two hundred and forty-seven trainees completed the module evaluation (56% response rate). As seen in Table 2, respondents very positively evaluated their own accomplishment of the learning objectives (highest accomplishment in Module 2 and lowest accomplishment achievement in Module 5).

**Table 2:** **Module learning objectives summary**

To what extent did this module enable you to meet the following learning objectives?				
Learning objectives:	N = #respondents who completed the evaluation	Completely accomplished	Partially accomplished	Did Not accomplish
Module 1	101	91.4%	6.9%	1.7%
Module 2	14	95.2%	4.8%	0%
Module 3	36	83.8%	14.8%	1.4%
Module 4	36	81.2%	17.4%	1.4%
Module 5	21	76.2%	21.4%	2.4%
Module 6	21	84.1%	14.8%	1.1%

In the section where trainees were asked if they were satisfied with different design aspects of the modules, more than 80% of the participants agreed or strongly agreed with most of the satisfaction items (see Additional file 2 for actual parameters). While the learners indicated that the median rating for the newness of the material was somewhat lower (3.64), the median rating for the remainder of the module design aspects were 4.11 or greater. Overall resident satisfaction was high and the material was judged useful.

B. Results from the qualitative analysis

The following six themes emerged from the thematic data analysis:

### Theme 1: Satisfaction with the experience and key elements learned

Most of the participants indicated that they were pleased with the learning experience and would incorporate what they learned into their medical practices. The following were the most frequently cited topics:

-  Food groups (nutrient rich food, fruits, vegetables, snacks, junk food, the importance of breakfast, portion sizes for toddler, evaluation of whole grains, juices in pediatric diet, sugary drinks, diets, and dietary guidelines).
-  Breast feeding (importance, benefits, contradictions, and obstacles)
-  Dehydration and rehydration techniques (ORT, TPN)
-  Obesity (definition of obesity, trends, and adolescent obesity)

In addition, some of the participants pointed out that they would utilize the knowledge on parental education about nutrition-rich foods and strategies to determine nutritional needs after hospital discharge in their practice.

### Theme 2: Speaker quality and content coverage

A majority of participants described the best feature of the modules as the quality of speakers with their focused delivery of speech although one participant made a comment that s/he found the lecturer's tone condescending.

Participants were highly satisfied with the scope and the quality of modular content, describing them as very educational, informational and relevant. Most of the comments also confirmed that the modules were well-focused, practical, and easy to follow with adequate information providing several key concepts which could be discussed with patients. One of the participants commented on the non-interactive modules saying: “I would like to have an ongoing CD of this lecture to run continuously in my office waiting room. Getting the message through to the parents is a big step.” While a few of the participants felt the interactive modules were too long, one participant commented: “…I also appreciated that it [the content] was broken into manageable chunks, as well as the way the slides were integrated in with video of the speaker, which allowed a connection with him, rather than listening to some disembodied voice.” In addition, most participants indicated that they appreciated the quizzes and questions in the interactive modules as they helped to solidify the content and to check understanding during the module completion.

### Theme 3: Graphics and visuals

Most participants made positive comments about the use of strong visual images, including graphics, charts, illustrations, formulas, as well as the questions and answers sections in the interactive modules. Participants also appreciated the interactive and engaging nature of the modules since their interaction progressed during completion of the module (e.g., choosing answers by clicking on the diagrams). In a few instances, participants compared their experience with the non-interactive modules with the interactive ones. This is illustrated by a participant’s comment regarding knowledge retention after comparing two types of modules, *“*the multiple video module is less enjoyable and allows me to retain less information than the interactive modules in the earlier sections.”

### Theme 4: Navigation

There was some variability in the design and execution of the modules. The navigational aspects of Module 3 were noted by 24% of the participants (P < 0.02 – data not shown) to be more problematic than Module 1. This was also supported by qualitative data as illustrated by this participant quote, “sometimes it was unclear when I was supposed to advance the slide. The other complaint would be that the music interlude at the start of each section was too long and a waste of time”.

A few participants found the introduction section of the interactive modules somewhat long and “resume” functions to be problematic at times. One participant commented on the navigation of the interactive module by saying, “Having to continually click next was a little annoying. I understand it is needed if there are interactive features, but I would have just liked a little more flow when there was nothing interactive to slow me down.” Additionally, the navigation narration and the automated voice reading the text on some of the pages were noted to be the least favorite aspects of the interactive modules.

### Theme 5: Technical issues

There were also some comments about the technical difficulties participants encountered such as launching the video (Module 3), video freezing at times, not loading the “Questions and Answers” section properly and computer compatibility.

### Theme 6: Trainees’ suggestions for improvement

The length of the modules was a concern for some of the participants. They also commented that they did not like an interactive activity which was about writing an email to a patient’s mother regarding a given case. An individual detected a typographical error. Another participant commented on a video saying, “…watching a video is not my best learning modality. I would have liked to have a PowerPoint or script to follow as well.” Other proposed suggestions for module improvements included:

-  More details on amino acids, lipids and formulas for inborn errors of metabolism
-  Links to patient information resources
-  PowerPoint presentation accompanying the video
-  Eliminating those interactive questions that were too simplistic
-  Unnecessary audio with some slides
-  More in-depth didactic coverage of the information in the newest U.S. dietary guidelines

In summary, thematic analysis of open-ended questions revealed that the learners

- were satisfied with their investment of time
- would utilize this knowledge in their clinical practice
- were very positive and had high satisfaction with the speakers
- appreciated the focused nature of the module and module content
- appreciated the graphics and the interactive engagement activities in the modules
- were at times confused by navigation details in some modules
- were sometimes frustrated by technical difficulties, most of which appeared to be dependent on the device used to access the module.

## Discussion

Our study provides a description and preliminary assessment of the PNS curriculum modules, including both interactive and non-interactive formats, and their impact on pediatric trainees’ engagement, knowledge acquisition and satisfaction. The results show that the integration of technology-mediated delivery of a nutrition curriculum can provide a rich learning experience to enhance trainees’ knowledge while maintaining a high level of participant satisfaction.

Both interactive and non-interactive formats provided knowledge gains with the interactive format resulting in higher resident satisfaction than the non-interactive format. The knowledge gains between pretest and posttest were similar for interactive and non-interactive format. Other studies have demonstrated increased knowledge gains and higher learner satisfaction with more interactivity in online education [17–19]. The interactive/non-interactive main effects revealed significant differences for Modules 1 and 5 (with interactivity higher) at both baseline and post-test. Our study detected only a modest or slight improvement, and this may have been due to limitations in sample size for all formats in all modules or just that the interactivity components used in this series were not sufficiently robust to deliver a learning advantage. In keeping with our hypothesis, the pre/post-test effect for each module demonstrated the expected outcome of significant knowledge gain. Calculating the Cohen’s effect size for the interactive/non-interactive main effect and the pre/post-test by interactive/non-interactive effect, it was clear that the effects were small. It is certainly possible that the effects will be greater with more modules completed or, again, that the advantages of the interactivity used here is modest at best. Better understanding of any self-selection effects (we have no method to assess why residents chose to complete the modules and format they did) on the impact of the different instructional formats will be important to assess in future studies.

The primary goal of PNS is to develop defined levels of nutrition knowledge and skills in resident physicians caring for children in order to improve their ability to deliver effective nutrition care in clinical practice. Completing one or two modules may create short-term knowledge gains, but may not produce long-term desired changes in behaviors. The relatively low number of correct knowledge-based scores at baseline confirms the results of similar studies that document low nutrition knowledge in pediatric residents [8, 20, 21]. What is unknown at this time is whether completing multiple modules will produce significant outcomes in clinical practice.

In the present study, responses to open-ended questions captured some technology issues that participants encountered during completion of the modules. Although the PNS modules were tested using many platforms, this user feedback emphasizes the need for us and others who pursue multi-platform online education, to investigate the broad array of technology access platforms and provide a troubleshooting guide to the participants. Given the wide variety of computers and users, the fact that only six technical issues were identified is notable and we do not know whether these technical problems occurred because of the users’ computer system or were related to other factors. Given that each instance was an isolated concern, it does not appear that these issues were derived from the specific modules.

We have noted the feedback regarding module length; it is not surprising that there is a variety of preferences on this learning aspect. Since there are more user control features in the interactive method, it remains clear that any interactive task requires longer viewing time than non-interactive engagement. The interactive PNS modules have a book-marking feature which allows a learner the option of stopping a session and returning to the last slide completed in the previous session at a later time. We are not aware if the user has utilized this feature. However, we have already discussed the design issues with the PNS working group and are considering designing future modules in a ‘mini-module’ format.

There has been a significant growth in physician use of online learning in recent years. This is partly due to preferences of many young learners, the effects of ACGME duty hour work restrictions, and improvements in educational technologies and learning platforms that provide more reliable and robust options. In 2010, Harris predicted that half of all medical continuing education will be online by 2016 [22]. The availability of the PNS online modules with 24-hour, 7 day-a-week access gives trainees more flexibility to expand their nutrition education. A meta-analysis study conducted by Cook et al. [23] showed that internet-based learning in healthcare professions has significant benefits on learners’ knowledge. Given further restrictions on hospital time and availability for standard lecture attendance, graduate medical education leaders recognize the increasing need for non-traditional instructional technologies. The PNS platform addresses these concerns and also meets the increasing preference and comfort for trainees with self-directed online education. An additional benefit of the PNS system is the ability to tie module completion to specific rotations and learning experiences, something that is more difficult to accomplish with live teaching presentations. We believe that the PNS will find its best use as a complement to bedside/patient based learning.

The importance of nutrition education is increasingly being supported by research. Our study showed that with the use of PNS modules, overall trainees’ short-term knowledge gain and satisfaction was high and the material was judged useful. This early experience with the PNS demonstrates how to harness the ability of the internet (e-learning) to improve medical knowledge in important medical domains such as pediatric nutrition.

### Limitations and future direction

Our study has several limitations. First, only a small number of trainees completed some of the modules. Second, our sample is a self-selected diverse group of trainees, residing in multiple regions of the US. Because these participants may be more interested and motivated in the topic area than other physicians in training, this may create a positive learning bias. Further, trainees self-selected into either interactive or non-interactive formats, which may have created a nutrition-ability bias for some module comparisons. We were not able to obtain specific demographic data (gender and age) of the trainees who completed these modules because this information is provided during program registration but not upon module completion. Future efforts will include demographic data collection during module completion and evaluation. In fact, the PNS has already been enhanced for this purpose.

We still have much to learn about best methods to integrate online modules into trainee training. Varied learner preferences will likely drive perceptions of different educational approaches. More importantly, there remains the need to translate and assess the educational impact of the PNS modules and their completion on clinical competency and practice outcomes. Thus, further research is needed in three areas:

1. How well long-term knowledge gains persist after both interactive and non-interactive learning activities.
2. How trainees apply the knowledge and skills learned through PNS to improve patient care outcomes.
3. How motivation and self-direction drives knowledge and skill acquisition in these adult learners.

Finally, an important next step is to investigate the ability of e-learning to improve trainees’ nutrition care and counseling skills (competency). There is much still to be done with this innovative educational resource.

## Conclusion

Good nutrition is essential to a child's wellbeing. Many studies have pointed out that nutritious food in childhood improves educational outcomes, lower risk for disease, reduce rates of childhood obesity, and enhance the mental and emotional health of our children [24–27]. Recognizing existing deficiencies in pediatric nutrition education, specifically poor knowledge and skill defects, the development of the PNS curriculum laid the foundation for online methods to develop the nutritional knowledge and skills relevant to clinical practice for pediatric trainees and practicing physicians.

Preliminary assessment of these widely accessible and interactive PNS modules demonstrates that the integration of technology-mediated delivery of nutrition curriculum provides rich learning environments which enhance trainees’ knowledge while maintaining a high level of participant satisfaction. The future life-long learning needs of trainees and physicians in practice can be well-served by continued development of such technology enhanced learning platforms as the PNS. Furthermore, this industry-supported collaborative project can be regarded as a model for technology-enhanced curricular innovation and education in other areas of medicine.

## Appendix 1

Sample case discussion

The case discussions used real life situations to force the learners to connect their newfound knowledge of nutrition information with the need for patient evaluation, including management skills. These discussions allow the learner to develop and practice problem-solving and decision making skills in a learner-centered environment. Each PNS module is equipped with a set of 3–4 case discussions that can be used independently by a trainee or by groups of trainees and can be completed with or without a faculty facilitator. By supplementing each module with case discussions, trainees are driven to not only acquire the knowledge, but to also synthesize, evaluate, and apply the information they have learned.

Example: Acute Diarrhea and Dehydration Case Discussion

An 8 m/o girl presents to the Emergency Department with a 2-day history of vomiting and diarrhea and has been having difficulty keeping down any fluids in the past 24 hours. Parents report that it has been difficult for them to determine how much urine output there has been because of the diarrhea. Her weight today is 11 kg and her vital signs are temperature is 37°C, HR 170, RR 40, and BP 75/48.

Initial Assessment:

1. What historical questions would you ask to help determine the severity of her dehydration?
2. What factors increase the risk of dehydration in infants?
3. If present, which physical exam findings would suggest moderate to severe dehydration?
4. What studies would you consider performing if she was found to have moderate to severe dehydration?
5. Explain why ORT is better than IV rehydration in most situations.
6. What are some contraindications to using Oral Rehydration Therapy?

Upon completion of your initial assessment, you determine that this young girl has severe dehydration and proceed to administer a bolus of intravenous fluids. Upon re-assessment, she is more responsive, crying more, has better eye contact, and has better perfusion. Her parents also report that she is thirsty and is more interested in drinking fluids. Her repeat vital signs are temperature of 37.1°C, HR 130, RR 29, and BP 90/69.

Secondary Assessment:

1. What additional historical questions would you ask to clarify the cause of her illness?
2. What additional laboratory testing would you consider performing?

Sample applied learning activities

These activities are designed based on David Kolb’s learning cycle and “active experimentation” that extends nutritional knowledge and theory into practice. The activities are intended to help the resident make a connection between what s/he has learned in the nutrition modules and how that knowledge can be used for real life patient issues. For each module there are approximately 5–7 different authentic patient situations which ask the learner to complete a specific task and reflect on his or her experience.

Conduct a survey of your clinic to gauge the clinic's cultural milieu regarding body image. Based on your findings create positive changes in your clinic to enhance a healthy body image among all children in your clinic.

- Look at the magazines in your continuity or outpatient clinic. What percentage of these magazines portray or discuss an unhealthy body image on the cover? On average, throughout the magazine, were the models large, average or thin? Repeat this same activity for any pictures or posters you may have in the clinic.
- Observe the weigh and measure process in the clinic. What is said about the weight after it is taken? Does this change depending on the gender of the child or adolescent? Is this communication different based on ethnicity or race?
- Locate the handouts your clinic provides for both a healthy body image and eating disorders. Reflect on these handouts and determine if gaps exist in the information provided either to parents or teens.
- Reflect on the messages you give children and parents when discussing weight and nutrition. Do you emphasize thinness or focus excessively on obesity?

## Electronic supplementary material

Additional file 1: **Modules for the pediatric nutrition series curriculum.** (DOC 29 KB)

Additional file 2: **Chart 1.** Learner Satisfaction with Modules. (DOC 26 KB)

## Abbreviations

PNS: Pediatric Nutrition Series
NIM: Nutrition in Medicine
NEPP: Nutrition Education for Practicing Physicians
ABP: American Board of Pediatrics
ORT: Oral Rehydration Therapy
TPN: Total Parenteral Nutrition.
