# Supplementary material for: Pediatric trainees’ engagement in the online nutrition curriculum: preliminary results
Source: BMC Med Educ. 2014 Sep 16;14:190. doi: 10.1186/1472-6920-14-190 (PMC4179838; doi:10.1186/1472-6920-14-190)
Supplement: Supplementary file 1 — Additional file 1: Modules for the pediatric nutrition series curriculum. (DOC 29 KB) [file 12909_2013_1021_MOESM1_ESM.doc]

Additional file 1

| Modules | Description |
| --- | --- |
| *Module 1:* 10 Things You Must Know About Nutrition | This module describes the fundamentals of high quality, age appropriate nutrition counseling. It covers key problem areas of child nutrition, reviews the importance of breakfast and defines the concept of "nutrient rich foods" compared with the "avoidance" message. |
| *Module 2:* Be Knowledgeable About Breastfeeding | This module reviews the importance of breastfeeding and obstacles to breastfeeding and helps guide the resident in advocating for breastfeeding in their institution. |
| *Module 3:* Dehydration and Rehydration Techniques | This module provides a review of GI physiology and acute diarrheal illnesses. Assessment methods and treatment modalities including oral rehydration and refeeding are addressed. |
| *Module 4:* Fluids, Glucose, Calcium & Parenteral Nutrition for the Neonate | This module addresses parenteral nutritional issues for premature infants. It explains the dynamics of fluid balance, develops an algorithm for neonatal hypoglycemia, formulates a TPN plan for a premature infant, and identifies common complications of TPN. |
| *Module 5:* Enteral Nutrition for Premature Infants | This module addresses enteral nutritional issues for premature infants including specific nutritional needs and formulas, as well as common feeding problems. |
| *Module 6:* Over-Nutrition in Teens: Obesity and the Metabolic Syndrome | This module addresses the topic of obesity, providing information on standard terminology and pathophysiology, as well as the clinical evaluation and management of the overweight and obese adolescent. |
