# Supplementary material for: Pediatric trainees’ engagement in the online nutrition curriculum: preliminary results
Source: BMC Med Educ. 2014 Sep 16;14:190. doi: 10.1186/1472-6920-14-190 (PMC4179838; doi:10.1186/1472-6920-14-190)
Supplement: Supplementary file 2 — Additional file 2: Chart 1. Learner Satisfaction with Modules. (DOC 26 KB) [file 12909_2013_1021_MOESM2_ESM.doc]

Additional file 2: Chart 1: Learner Satisfaction with Modules

*Median scores calculated based on grouping data into class intervals

Learner satisfaction parameters

Abbreviated Parameter Actual Parameter

Interesting and enjoyable The module was interesting and enjoyable.

Clear goals Learning goals for the module were clearly stated.

Written clearly The module was clearly written.

Appropriate detail The module incorporated an appropriate level of detail.

Relevant materials The materials presented in this module were relevant to

the presented topic.

Extended my knowledge The materials presented in this module helped me extend

my knowledge on the presented topic.

Easy to understand The module materials were easy to understand.

New material The module materials were mostly new to me.

Well organized The module materials were well organized.

Reasonable completion time The amount of time it took to complete the module was reasonable.
